# The Impact of Internet Addiction and Job Satisfaction on Mental Health Symptoms among a Sample of Portuguese Workers

**DOI:** 10.3390/ijerph18136943

**Published:** 2021-06-29

**Authors:** Henrique Pereira, Gergely Fehér, Antal Tibold, Graça Esgalhado, Vítor Costa, Samuel Monteiro

**Affiliations:** 1 Department of Psychology and Education, Faculty of Social and Human Sciences, University of Beira Interior, Pólo IV, 6200-209 Covilhã, Portugal; mgpe@ubi.pt (G.E.); vitormvc@ubi.pt (V.C.); smonteiro@ubi.pt (S.M.); 2 Research Centre in Sports Sciences, Health Sciences and Human Development (CIDESD), 5001-801 Vila Real, Portugal; 3 Centre for Occupational Medicine, Medical School, University of Pécs, 7624 Pécs, Hungary; feher.gergely@pte.hu (G.F.); tibold.antal@pte.hu (A.T.); 4 Institute of Cognitive Psychology, Human and Social Development (IPCDHS), 3000-115 Coimbra, Portugal; 5 NECE—Research Center in Business Science, Faculty of Social and Human Sciences, University of Beira Interior, Pólo IV, 6200-209 Covilhã, Portugal

**Keywords:** internet addiction, job satisfaction, mental health

## Abstract

Not many studies assessing the impact of internet addiction (IA) and job satisfaction (JS) on mental health symptoms (MHS) among active workers exist. Therefore, the purpose of this study was as follows: (a) to assess the presence of criteria for IA among a sample of active workers; (b) to analyze differences in IA, JS and MHS, by gender; (c) to analyze association levels among IA, JS, and MHS; and (d) to determine the predictive effect of IA and JS on MHS. In total, 1064 participants (*M_age_* = 40.66; *SD* = 12.02) completed a survey containing four categories of measures: demographic information, internet addiction, job satisfaction, and mental health symptoms (anxiety and depression). Results showed a presence of 13.3% for IA among the sample. Male participants showed higher scores of IA and JS but lower scores of overall MSH than female participants did. Significant positive correlations were found between overall IA and MHS, and significant negative correlations were found between IA and JS, and MHS and JS. Hierarchical linear regression analysis showed that strong predictors of MHS were age (being older), gender (being female), not having enough economic funds, being unsatisfied with the leadership in the job, being unsatisfied with the nature of the job, and having higher scores in salience and excessive use regarding IA. In conclusion, addiction to internet technology is a risk factor with implications for occupational satisfaction and mental health.

## 1. Introduction

Internet technology has irrefutably impacted human behavior in several core domains of our experience. The potential adverse effects of internet exposure on human health have emerged as a major global concern [1], including problematic or overuse of internet services and internet addiction, the prevalence of which has been increasing over time [2]. These effects have been mostly studied in specific phases of the lifespan (i.e., adolescence and early adulthood) and seem conditioned by environmental and systemic circumstances facilitating or inhibiting factors [3].

Following a biopsychosocial paradigm that explains health as the interactions of a person’s genetic and psychological make-up with the environment, addictions are a part of this biopsychosocial process [4,5,6]. Internet addiction (IA) has been increasingly conceptualized as a mental health disorder [7], since several studies have demonstrated that IA shares similar symptomatology to that of addictive disorders [8]. Despite the limited data about the prevalence of IA among both adults in general and active workers in particular, two meta-analytic studies have reported prevalence rates of IA from 1996 to 2018 [2,9]. The analysis of 80 [9] and 113 [2] epidemiologic studies revealed consistent differences between IA prevalence in different regions (higher in eastern and lower in western countries) [4] with an estimated overall prevalence of 7.02% [2]. Further research has suggested that IA is associated with relevant psychological impairment, such as psychopathology, serious mental illness including depression and anxiety [10], and suicidality [10]. Common significant predictors of IA include age (being < 35 years), being male, using the internet > 5 h daily, having no children, having secondary employment [11], taking illicit drugs, being diabetic [12], dysfunctional emotional regulation [13], loneliness [14], lower levels of self-control and higher levels of foreclosed and diffuse identity [15], low education among parents, lifetime tobacco use [16], social connectedness, social media addiction, digital game addiction and smartphone addiction [17], low self-esteem [18], and low emotional intelligence [19].

Several studies have also demonstrated the negative impact of IA on workers’ performances. The nature of the work people do in the 21st century is heavily dependent on internet technologies, which could negatively affect productivity [20] and job satisfaction [21]. In fact, job satisfaction (JS) can be seen as a combination of psychological, physiological, and environmental circumstances that cause a person to be satisfied with their job, and it is mediated by a myriad of factors, such as organizational commitment and job involvement [22], burnout, turnover intention, job demands and job resources [23], job attitudes [24], self-efficacy [25,26], and resilience [27]. Since most people spend many hours of their day working, JS is a crucial factor to promote both occupational health and well-being in general and occupational mental health in particular. Several studies have suggested, regardless of work contexts, the existence of a significant positive relationship between JS and satisfaction with life [28] as well as psychological and social well-being [29]. On the other hand, having lower levels of JS is usually associated with burnout and turnover intention [30]; job characteristics, such as a lack of autonomy or a low perceived cultural novelty [31]; and workplace stressors such as abusive supervision [32].

Not many studies explore the association between IA and JS on mental health symptoms, especially among active workers. Portuguese data on this association are scarce and are usually conducted with adolescents and/or students. Nevertheless, some studies have found a prevalence of IA of 19% among Portuguese adolescents, associated with a male gender, use of social networks (mainly Twitter and Instagram), self-perceived sleep problems, initial and middle insomnia, and excessive daytime sleepiness [33].

Even though IA describes a term applied in the study of different technology addiction problems, we chose to focus on this construct because it is more generalized in several domains (clinical, epidemiological, and psychiatric), and despite some controversies, it represents a rich and necessary field from a scientific perspective with relevant implications for the understanding of overall quality of life of active workers. Studies exploring JS determinants from multi-occupational workers are also scarce and limited to its association with burnout, work engagement, and work-related quality of life [34]. No studies concerning the impact of IA and JS on mental health symptoms (MHS) were found, and, therefore, the aim of our study was as follows: (a) to detect the presence of criteria for internet addiction among a sample of active workers; (b) to analyze differences in IA, JS, and MHS by gender; (c) to analyze association levels among IA, JS, and MHS; and (d) to determine the predictive effect of IA and JS on MHS. We believe that our results will contribute to a growing body of international scientific knowledge committed to improving the reliability of data generated in the field of IA, JS, and MHS, which is particularly relevant in the era of globalization and corporate internationalization.

## 2. Materials and Methods

### 2.1. Measurement Instruments

The survey included four categories of questions/measures: demographic information, internet addiction, job satisfaction, and mental health symptoms (anxiety and depression).

Demographic Information. Items included age, gender, educational attainment, place of residence, marital status, professional status, having children, sector of activity, having enough financial resources, and overall health self-assessment.

Internet Addiction. The Portuguese version of the Internet Addiction Test (IAT) was used [35]. It comprises 20 items, each of which is rated on a five-point Likert scale: ‘rarely or never’ (1), ‘occasionally’ (2), ‘frequently’ (3), ‘very often’ (4), and ‘always’ (5). The test measures the extent of a person’s involvement with the internet and classifies addictive behavior. To obtain the test’s overall score, one needs to sum the scores for all responses provided by participants. Presence of IA was calculated according to Young’s most recent criteria [35]: normal range (0–30); mildly addicted (31–49); moderately addicted (50–79); and severely addicted (80–100). For the calculation of the percentage of participants with IA, a sum of the participants in the moderately and severely addicted ranges (i.e., 50–100) was considered. Furthermore, factor analysis of the IAT revealed six dimensions: salience, excessive use, neglecting work, anticipation, lack of control, and neglecting social life. Cronbach’s alpha revealed high reliability levels for the IAT (0.92).

Job Satisfaction. The Portuguese version of the Job Satisfaction Scale (JSS) [36] was used in this study. It comprises 15 items that assess job satisfaction related to work colleagues, salary, leadership, nature of the job, and career promotions. Participants answered the questions based on their self-assessment of job satisfaction using a 7-point scale ranging from 1 (not satisfied at all) to 7 (completely satisfied). The total score was calculated using a simple mean score of all items, with higher scores indicating higher levels of job satisfaction. Cronbach’s alpha revealed high reliability levels for the JSS (0.93).

Mental Health Symptoms (Depression and Anxiety). Participants’ perceived mental health symptoms (MHS) were measured using the anxiety and depression sub-scales of the Portuguese version of the Brief Symptom Inventory (BSI-18) [37,38]. The BSI-18 is an 18-item scale that measures depression, anxiety, and somatic symptoms in non-clinical populations and is helpful to screen MHS. For the purpose of this study, only the depression and anxiety subscales were used since they represent the most prevalent mental health problems among the general population. Participants answered questions based on their self-assessment of mental symptoms using a 5-point scale ranging from 0 (never) to 4 (always). The total score was calculated using a simple mean score, with higher scores indicating more symptoms. Cronbach’s alphas revealed high reliability levels for each factor (0.89 for depression; 0.87 for anxiety). To produce an overall measure of mental health symptoms, the authors computed depression and anxiety scores into one single variable using the mean scores.

### 2.2. Procedures

This research was carried out through an online survey that was available between October and December 2018. Participation was voluntary, and participants were referred to a secure linked website created specifically for the purpose of this research. The first page of the questionnaire explained the objectives of the study and informed participants about how to fill it in, how to withdraw from the study, and how to contact the authors for more information. They were also asked to read and explicitly agree to an informed consent waiver.

A total of 6000 notifications were sent, and 1064 participants responded (17.7% response rate). The questionnaire was disseminated through social networks, random e-mails and mailing lists, and discussion forums, respecting all the ethical principles of research in psychology (informed consent, confidentiality, and anonymity). Neither rewards nor other incentives were offered. Inclusion criteria, validated during and after data collection, included the following: being older than 18 years of age; being a Portuguese native speaker; and being actively engaged in the workforce via being employed, self-employed, or working and studying. Part-time workers were excluded from this study. Ethical approval for this study was granted by the Ethics Committee of the University of Beira Interior, Portugal.

### 2.3. Data Analysis

Descriptive statistics were performed to describe the sample (mean, standard deviation, frequencies, and percentages). Student t-tests were conducted to evaluate differences between comparison groups. To assess the association between IA, JS and MHS, Pearson correlation coefficients were conducted. Finally, a hierarchical linear regression analysis was conducted to examine the effects of independent variables (sociodemographic variables, IA and JS) on the dependent variable (MHS). All analyses were performed using software IBM SPSS (Statistical Package for the Social Sciences, Armonk, NY, USA), version 27.

## 3. Results

In total, 1064 Portuguese workers between 19 and 74 years of age participated in this study (mean = 40.66; *SD* = 12.02); among them, 53% identified as female and 47% as male. The majority claimed to be employed (62.5%) or self-employed (20.3%), possessed a university education (87.6%), and had sufficient economic funds for everyday subsistence (58.2%). In addition, 44.3% said they were married, 55.8% lived in large urban environments, 53.3% worked in the private sector, and 53.3% had no children. The overall health self-assessment was good or very good (58.5%). Table 1 presents the sample’s sociodemographic characteristics in further detail.

The results regarding the presence of IA showed that 42.3% of participants fell in the normal range (i.e., show no addiction), 44.2% complied with the criteria for mild addiction, 12.4% were moderately addicted, and 0.9% showed criteria for the presence of severe IA. Considering moderately and severely addicted percentages together, the results showed a total presence of criteria for IA of 13.3%. Table 2 shows the results for participants’ internet addiction, mental health, and job satisfaction levels. On average, participants scored 1.80 (*SD =* 0.64) for overall internet addiction, 2.13 (*SD =* 0.86) for mental health symptoms, and 4.47 (*SD =* 1.10) for overall job satisfaction. Anticipation and excessive use were the most prevalent dimension for internet addiction, whereas satisfaction with salary and career promotion scored lower for job satisfaction. Participants’ general mental health perceptions were also relatively low, as shown by an average score of 2.13 (*SD =* 0.86) on the general mental symptoms self-assessment.

We also analyzed differences in internet addiction, mental health symptoms, and job satisfaction levels by genders. The results showed statistically significant differences (*p* < 0.05) for overall internet addiction (t _(1044)_ = 4.162; *p* < 0.001), overall mental health symptoms (t _(1056)_ = −4.656; *p* < 0.001), and overall job satisfaction (t _(1028)_ = 2.987; *p* = 0.003), indicating that male participants showed higher scores of internet addiction and job satisfaction but lower scores of overall mental health symptoms than female participants did (see Table 3 for further details on different categories for each variable).

A correlation matrix was created using overall variables to assess the levels of association among internet addiction, mental health symptoms, and job satisfaction. As displayed in Table 4, significant correlations were found between overall internet addiction and mental health symptoms (*r* = 0.238; *p* < 0.001); internet addiction and job satisfaction (*r* = −0.063; *p* < 0.05); overall mental health symptoms and overall job satisfaction (*r* = −0.345; *p* < 0.001); and overall levels of internet addiction, overall levels of mental health symptoms, overall levels of job satisfaction, and age (*r* = −0.171; *p* < 0.001; *r* = −0.195; *p* < 0.001; *r* = 0.173; *p* < 0.001).

Finally, we carried out a hierarchical linear regression analysis to assess the effects that sociodemographic variables, internet addiction, and job satisfaction have on the mental health symptoms of the sample. The variables “age”, “gender”, and “professional status” were added in the first block (Model I). Job satisfaction variables were added in the second block (Model II). Internet addiction variables were added in the third block (Model III). The first block of analysis explained 5% of the variance of mental health symptoms, the second block explained 15%, and the third block explained 20%. Thus, as shown in Table 5, strong predictors of mental health symptoms were age (being older), gender (being female), being unsatisfied with the leadership in the job, being unsatisfied with the nature of the job, and having higher scores in salience and excessive use regarding internet addiction.

## 4. Discussion

The results showed a reduced presence of severe internet addiction in 0.9% of the sample. However, moderately addicted and severely addicted grouped together reached 13.3% of the current sample, which is higher than the prevalence found in previous research [39,40,41]. Regarding gender differences, results drawn from the current sample reinforce that males are more prone to internet addiction than female participants are [42], with statistically significant differences between both groups for all IA dimensions, apart from anticipation. Female participants in our sample reported depression and anxiety symptoms more frequently when compared to male participants, with statistically significant differences, which reinforces previous research [42]. Concerning job satisfaction, male participants showed higher satisfaction than females did. This result goes against the so-called paradox of the contented female worker but is aligned with up-to-date analysis on the topic [43], which considers the effects of the gender gap.

Aligning with recent research [10,42,44], a significant correlation was found between internet addiction and mental health symptoms in the present study. Additionally, IA dimensions of salience and excessive use were significant predictors of mental health symptoms. That is, participants who are heavy internet users stay online longer than intended, and individuals to whom internet represents a critical life dimension tend to experience anxiety and depression symptoms more frequently. The significant negative association between internet addiction and job satisfaction found in the current study contributes to a scarce body of work that explores these variables in adult working samples [45], showing that an increase in IA is associated with a decrease in JS.

Job satisfaction is also negatively correlated to MHS, showing that when an increase in job satisfaction is observed in our sample, depression and anxiety symptoms tend to decrease. The reported results demonstrate the potential protector effect of job satisfaction on health-considered dimensions, as verified in previous studies [46]. Additionally, hierarchical regression results showed that JS dimensions of satisfaction with both leadership and job nature were statistically significant negative predictors of MHS. This result shows that workers who are more satisfied with their leaders’ characteristics (e.g., competency) and with the inherent characteristics of the job show fewer symptoms of depression and anxiety. In fact, having a meaningful job and being satisfied with the job are important predictors of lower depression and anxiety [47].

As a practical implication, our results suggest that IA is a relevant problem that should be assessed (and, if necessary, intervened) during adulthood, demystifying the idea that this type of addiction is mainly relevant in phases of the life cycle without free will, such as adolescence or childhood. Furthermore, regarding job satisfaction indicators, our results suggest that policies and practices that emphasize job satisfaction, oriented to enrich tasks and improve the interaction between the leader and the led, may generate improvements in health levels, reducing anxiety and depression in particular.

### 4.1. Limitations

This study is not without limitations, since it follows a cross-sectional design, which limits the generalization of its results’ focus on various constructs of self-assessment that tend to change over time. Additionally, some limitations to the results’ generalization are associated with the data collection process and outcomes, which do not allow us to assume population representativeness. Regarding the prevalence of internet addiction, the lack of a precise diagnostic criteria [35] hinders thorough comparisons between different studies. In this study, the more recently proposed cut-off values were used [46], and, thus, severe internet addiction was considered for participants with a score between 80 and 100. However, due to current research still using older guidelines [10,43], in which addictive use of internet was considered for participants with a 70–100 score, the criteria for severe internet addiction cannot be compared in these cases.

### 4.2. Future Research

The literature is still scarce on the topic of IA among working samples. Despite the contribution of the present paper, future research should continue to explore these themes while collecting additional information regarding cross-national variations and human heterogeneity at macro/environmental, organizational, and occupational settings as well as the micro/individual level across the lifespan. Changes in work (e.g., teleworking) related to the COVID-19 pandemic can be explored in its relation to IA, JS, and MHS. Thus, multivariate data analysis that allows the testing of multiple relationships simultaneously, such as SEM, and longitudinal studies aiming to observe variables, dynamics, and changes and further develop the evaluated constructs may be relevant in the future.

## 5. Conclusions

This research analyzed potential predictors of mental health indicators related to specific technology use/abuse and indicators of occupational satisfaction. In particular, the present study aimed to detect IA criteria among a sample of professional active Portuguese adults. Analyzing gender differences in IA, JS, and MHS, the association between the three variables of IA, JS, and MHS, and the predictive effect of IA and JS on MHS, we may conclude that a type of dysfunctional human–technology interaction, such as internet addiction, was not neutral for mental health symptoms. We may also conclude that, in adulthood, internet overuse and occupational satisfaction were inversely associated. This reflects the potential negative interaction between the “digital” world and the “real” world, enhancing an existential gap or duality that is potentially dangerous in terms of mental health risk. In conclusion, addiction to internet technology is a risk factor with implications for occupational satisfaction and mental health.

Finally, we reinforce the importance of the job satisfaction variable for active adults’ mental health. This research evidenced the occupational satisfaction potential for reducing anxiety and depression symptoms, and, therefore, job satisfaction could be a protective factor of the functioning of mental health. Globally, this research warns against the negative interaction between personal and professional life dimensions and the emergence of “new” forms of harmful technological use patterns or addictions at a development stage when (adult) individuals are, in theory, seen as fully autonomous and possessors of free will in their life choices. These negative interactions (“digital” vs. “real”; “personal” vs. “professional”) could affect adult adaptability to an increasingly volatile, uncertain, complex, and ambiguous world, and could be an essential domain in both the understanding of the contemporary adulthood phase and the rethinking of andragogic politics and practices.

## Figures and Tables

**Table 1:** Sociodemographic characteristics (*N* = 1064; *M_age_* = 40.66; *SD* = 12.02).

Variable	Categories	*N*	*%*
Gender	Male	500	47
	Female	564	53
Educational Attainment	Middle school	23	2.1
	Secondary school	110	10.3
	University	931	87.6
Place of Residence	Small rural environment	49	4.6
	Large rural environment	43	4.0
	Small urban environment	378	35.6
	Large urban environment	594	55.8
Marital Status	Single	302	28.4
	Married	468	44.3
	De facto union	111	10.3
	Widower	11	1.0
	Dating	76	7.1
	Divorced/separated	96	8.9
Professional Status	Working student	183	17.2
	Employed	665	62.5
	Self-employed	216	20.3
Children	Yes	497	46.7
	No	567	53.3
Sector of Activity	Public sector	497	46.7
	Private sector	567	53.3
Enough Financial Resources	Yes	619	58.2
	No	445	41.8
Overall Health Self-Assessment	Bad or very bad	116	10.9
	Average	326	30.6
	Good or very good	622	58.5

**Table 2:** Internet addiction, mental health symptoms, and job satisfaction levels.

Variable	Categories	Mean	Median	*SD*	Min	Max
Internet Addiction	Salience	1.56	1.20	0.71	1.00	5.00
	Excessive use	1.99	1.80	0.70	1.00	5.00
	Neglect work	1.67	1.33	0.88	1.00	5.00
	Anticipation	2.45	2.50	1.14	1.00	5.00
	Lack of control	1.80	1.67	0.83	1.00	5.00
	Neglect social life	1.55	1.50	0.72	1.00	5.00
	Overall internet addiction	1.80	1.60	0.64	1.00	5.00
Mental Health Symptoms	Anxiety symptoms	2.42	2.00	0.96	1.00	5.00
	Depressive symptoms	1.84	2.00	0.98	1.00	5.00
	Overall health symptoms	2.13	2.00	0.86	1.00	5.00
Job Satisfaction	Colleagues	4.9	5.00	1.20	1.00	7.00
	Salary	3.8	4.00	1.58	1.00	7.00
	Leadership	4.44	4.67	1.34	1.00	7.00
	Job’s nature	4.95	5.00	1.25	1.00	7.00
	Career promotions	3.87	4.00	1.50	1.00	7.00
	Overall job satisfaction	4.47	4.53	1.10	1.00	7.00

Note: The mean was found by adding all the scores together and dividing by the number of items in each set. The median was found by ordering the set from lowest to highest and finding the exact middle.

**Table 3:** Internet addiction, mental health symptoms, and job satisfaction levels between male and female participants.

Variables	Categories	Gender	*M*	*SD*	*t*(*df*)	*p*
Internet Addiction	Salience	Male	1.64	0.80	3.462(1043)	0.001 *
		Female	1.49	0.60		
	Excessive use	Male	2.07	0.73	3.160(1044)	0.002 *
		Female	1.93	0.68		
	Work neglect	Male	1.81	0.98	4.980(1041)	0.000 **
		Female	1.54	0.76		
	Anticipation	Male	2.51	1.17	1.687(1043)	0.092
		Female	2.39	1.10		
	Lack of control	Male	1.88	0.87	3.097(1043)	0.002 *
		Female	1.72	0.79		
	Social life neglect	Male	1.68	0.79	5.969(1043)	0.000 **
		Female	1.42	0.62		
	Overall internet addiction	Male	1.88	0.70	4.162(1044)	0.000 **
		Female	1.72	0.56		
Mental Health Symptoms	Anxiety symptoms	Male	2.29	0.92	−3.866(1053)	0.000 **
		Female	2.52	0.97		
	Depression symptoms	Male	1.69	0.91	−4.553(1052)	0.000 **
		Female	1.96	1.00		
	Overall mental health symptoms	Male	2.00	0.81	−4.656(1056)	0.000 **
		Female	2.24	0.87		
Job Satisfaction	Colleagues	Male	4.99	1.20	2.247(1027)	0.025 *
		Female	4.83	1.18		
	Salary	Male	3.95	1.55	2.947(1021)	0.003 *
		Female	3.66	1.58		
	Leadership	Male	4.49	1.35	1.095(1019)	0.274
		Female	4.39	1.31		
	Nature of job	Male	5.06	1.22	2.796(1021)	0.005 *
		Female	4.84	1.26		
	Career promotions	Male	4.05	1.50	3.459(1018)	0.001 *
		Female	3.72	1.50		
	Overall job satisfaction	Male	4.58	1.09	2.987(1028)	0.003 *
		Female	4.38	1.08		

* *p* < 0.05; ** *p* < 0.001.

**Table 4:** Correlation matrix.

	1	2	3	4
1—Overall Levels of Internet Addiction	-			
2—Overall Levels of Mental Health Symptoms	0.238 **	-		
3—Overall Levels of Job Satisfaction	−0.063 *	−0.345 **	-	
4—Age	−0.171 **	−0.195 **	0.173 **	-

* *p <* 0.05; ** *p <* 0.001.

**Table 5:** Hierarchical linear regression analysis predicting mental health symptoms (*N* = 1064).

	Model I				Model II			Model III		
	*B*	*SE B*	*Β*		*B*	*SE B*	*β*	*B*	*SE B*	*β*
Age	−0.012	0.002	−0.171 **		−0.009	0.002	−0.126 **	−0.007	0.002	−0.091 *
Gender	0.0191	0.054	0.111 **		0.164	0.052	0.095 *	0.227	0.052	0.132 **
Professional status	−0.029	0.047	−0.021		0.007	0.045	0.005 **	0.022	0.044	0.015
JS—Colleagues					−0.031	0.028	−0.043	−0.029	0.027	−0.040
JS—Salary satisfaction					−0.037	0.026	−0.068	−0.044	0.026	−0.081
JS—Leadership satisfaction					−0.063	0.033	−0.098	−0.067	0.032	−0.104 *
JS—Job nature					−0.129	0.027	−0.188 **	−0.098	0.027	−0.143 **
JS—Career promotion					0.002	0.026	0.003	−0.009	0.026	−0.016
IA—Salience								0.159	0.055	0.131 *
IA—Excessive use								0.131	0.065	0.108 *
IA—Work neglect								0.002	0.038	0.002
IA—Anticipation								0.022	0.029	0.029
IA—Lack of control								−0.042	0.046	−0.041
IA—Social life neglect								0.035	0.041	0.030
*R* ^2^		0.050				0.153			0.202	
*F*		17.280 **				21.985 **			17.432 **	

* *p <* 0.05; ** *p <* 0.001. NOTE: JS—job satisfaction; IA—internet addiction; Gender: male/female; Professional status: working student/employed/self-employed.

## Data Availability

The data presented in this study are available upon request.

## References

[1] Saunders J.B., Hao W., Long J., King D., Mann K., Fauth-Bühler M., Rumpf H.-J., Bowden-Jones H., Rahimi-Movaghar A., Chung T. (2017). Gaming disorder: Its delineation as an important condition for diagnosis, management, and prevention. J. Behav. Addict..

[2] Pan Y.-C., Chiu Y.-C., Lin Y.-H. (2020). Systematic review and meta-analysis of epidemiology of internet addiction. Neurosci. Biobehav. Rev..

[3] Ostovar S., Allahyar N., Aminpoor H., Moafian F., Nor M.B.M., Griffiths M.D. (2016). Internet Addiction and its Psychosocial Risks (Depression, Anxiety, Stress and Loneliness) among Iranian Adolescents and Young Adults: A Structural Equation Model in a Cross-Sectional Study. Int. J. Ment. Health Addict..

[4] Kimberley M., Zittel A.C.S.W., Shawn Lawrence C.S.W., Wodarski J.S. (2002). Biopsychosocial Model of Health and Healing. J. Hum. Behav. Soc. Environ..

[5] Wade D.T., Halligan P.W. (2017). The biopsychosocial model of illness: A model whose time has come. Clin. Rehabil..

[6] Griffiths M.D. (2005). A “components” model of addiction within a biopsychosocial framework. J. Subst. Use.

[7] Young K., Abreu C. (2011). Internet Addiction: A Handbook and Guide to Evaluation and Treatment.

[8] Vigna-Taglianti F., Brambilla R., Priotto B., Angelino R., Cuomo G., Diecidue R. (2017). Problematic internet use among high school students: Prevalence, associated factors and gender differences. Psychiatry Res..

[9] Cheng C., Li A.Y.-L. (2014). Internet Addiction Prevalence and Quality of (Real) Life: A Meta-Analysis of 31 Nations Across Seven World Regions. Cyberpsychol. Behav. Soc. Netw..

[10] Guo W., Tao Y., Li X., Lin X., Meng Y., Yang X., Wang H., Zhang Y., Tang W., Wang Q. (2020). Associations of Internet Addiction Severity With Psychopathology, Serious Mental Illness, and Suicidality: Large-Sample Cross-Sectional Study. J. Med. Internet Res..

[11] Tóth G., Kapus K., Hesszenberger D., Pohl M., Kósa G., Kiss J., Pusch G., Fejes É., Tibold A., Feher G. (2021). Prevalence and Risk Factors of Internet Addiction among Hungarian High School Teachers. Life.

[12] Toth G., Kapus K., Hesszenberger D., Pohl M., Kosa G., Kiss J., Pusch G., Fejes E., Tibold A., Feher G. (2021). Internet Addiction and Burnout in A Single Hospital: Is There Any Association?. Int. J. Environ. Res. Public Health.

[13] Yildiz M.A. (2017). Emotion regulation strategies as predictors of internet addiction and smartphone addiction in adolescents. J. Educ. Sci. Psychol..

[14] Tras Z. (2019). Internet Addiction and Loneliness as Predictors of Internet Gaming Disorder in Adolescents. Educ. Res. Rev..

[15] Agbaria Q., Bdier D. (2021). The Role of Self-Control and Identity Status as Predictors of Internet Addiction among Israeli-Palestinian College Students in Israel. Int. J. Ment. Health Addict..

[16] Mellouli M., Zammit N., Limam M., Elghardallou M., Mtiraoui A., Ajmi T., Zedini C. (2017). Prevalence and Predictors of Internet Addiction among College Students in Sousse, Tunisia. J. Res. Health Sci..

[17] Savci M., Aysan F. (2017). Technological addictions and social connectedness: Predictor effect of internet addiction, social media addiction, digital game addiction and smartphone addiction on social connectedness. Dusunen Adam J. Psychiatry Neurol. Sci..

[18] Seabra L., Loureiro M.J.D.S., Pereira H., Monteiro S., Afonso R.M., Esgalhado G. (2017). Relationship Between Internet Addiction and Self-Esteem: Cross-Cultural Study in Portugal and Brazil. Interact. Comput..

[19] Saraiva J., Esgalhado G., Pereira H., Monteiro S., Afonso R.M., Loureiro M.J.D.S. (2018). The Relationship Between Emotional Intelligence and Internet Addiction Among Youth and Adults. J. Addict. Nurs..

[20] Shrivastava A., Sharma M.K., Marimuthu P. (2018). Internet addiction at workplace and it implication for workers life style: Exploration from Southern India. Asian J. Psychiatry.

[21] Venkatesh V., Sykes T., Chan F., Thong J., Hu P. (2019). Children’s Internet Addiction, Family-to-Work Conflict, and Job Outcomes: A Study of Parent-Child Dyads. MIS Q..

[22] Ćulibrk J., Delić M., Mitrović S., Ćulibrk D. (2018). Job Satisfaction, Organizational Commitment and Job Involvement: The Mediating Role of Job Involvement. Front. Psychol..

[23] Scanlan J.N., Still M. (2019). Relationships between burnout, turnover intention, job satisfaction, job demands and job resources for mental health personnel in an Australian mental health service. BMC Health Serv. Res..

[24] Judge T.A., Weiss H.M., Kammeyer-Mueller J.D., Hulin C.L. (2017). Job attitudes, job satisfaction, and job affect: A century of continuity and of change. J. Appl. Psychol..

[25] De Simone S., Planta A., Cicotto G. (2018). The role of job satisfaction, work engagement, self-efficacy and agentic capacities on nurses’ turnover intention and patient satisfaction. Appl. Nurs. Res..

[26] Capone V., Petrillo G. (2020). Mental health in teachers: Relationships with job satisfaction, efficacy beliefs, burnout and depression. Curr. Psychol..

[27] Zheng Z., Gangaram P., Xie H., Chua S., Ong S.B.C., Koh S.E. (2017). Job satisfaction and resilience in psychiatric nurses: A study at the Institute of Mental Health, Singapore. Int. J. Ment. Health Nurs..

[28] Tadić M., Bakker A.B., Oerlemans W.G. (2013). Work happiness among teachers: A day reconstruction study on the role of self-concordance. J. Sch. Psychol..

[29] Capone V., Petrillo G., Romano A. (2013). La soddisfazione lavorativa e per la vita di medici e infermieri ospedalieri: Relazioni con il senso di appartenenza all’azienda ospedaliera, le percezioni di efficacia collettiva e il sostegno sociale percepito. Psicol. Della Salut..

[30] Labrague L., McEnroe-Petitte D., Gloe D., Tsaras K., Arteche D., Maldia F. (2017). Organizational politics, nurses’ stress, burnout levels, turnover intention and job satisfaction. Int. Nurs. Rev..

[31] Stoermer S., Lauring J., Selmer J. (2020). Job characteristics and perceived cultural novelty: Exploring the consequences for expatriate academics’ job satisfaction. Int. J. Hum. Resour. Manag..

[32] Peltokorpi V., Ramaswami A. (2021). Abusive supervision and subordinates’ physical and mental health: The effects of job satisfaction and power distance orientation. Int. J. Hum. Resour. Manag..

[33] Ferreira C., Ferreira H., Vieira M.J., Costeira M., Branco L., Dias A., Macedo L. (2017). Epidemiology of Internet Use by an Adolescent Population and its Relation with Sleep Habits. Acta Med. Port..

[34] Sinval J., Marôco J. (2020). Short Index of Job Satisfaction: Validity evidence from Portugal and Brazil. PLoS ONE.

[35] Pontes H.M., Patrão I.M., Griffiths M.D. (2014). Portuguese validation of the Internet Addiction Test: An empirical study. J. Behav. Addict..

[36] Rueda F.J.M. (2015). Análise fatorial confirmatória da Escala de Satisfação no Trabalho nas versões de 25 e 15 itens. Rev. Psicol. Organ. Trab..

[37] Derogatis L.R. (2001). BSI 18–Brief Symptom Inventory 18: Administration, Scoring, and Procedures Manual.

[38] Nazaré B., Pereira M., Canavarro M.C. (2017). Avaliação breve da psicossintomatologia: Análise fatorial confirmatória da versão portuguesa do Brief Symptom Inventory 18 (BSI 18). Análise Psicológica.

[39] Bisen S.S., Deshpande Y. (2020). Prevalence, predictors, psychological correlates of internet addiction among college students in India: A comprehensive study/Hindistan’da universite ogrencileri arasinda yayginlik, ongoruculer, internet bagimliliginin psikolojik bagintilari: Kapsamli bir calisma. Anadolu Psikiyatr. Derg..

[40] Bruno A., Scimeca G., Cava L., Pandolfo G., Zoccali R.A., Muscatello M.R. (2014). Prevalence of Internet Addiction in a Sample of Southern Italian High School Students. Int. J. Ment. Health Addict..

[41] Christakis D.A., Moreno M.M., Jelenchick L., Myaing M.T., Zhou C. (2011). Problematic internet usage in US college students: A pilot study. BMC Med..

[42] Puente A.C.F., Sánchez-Sánchez N. (2021). How Gender-Based Disparities affect Women’s Job Satisfaction? Evidence from Euro-Area. Soc. Indic. Res..

[43] Al Shawi A.F., Hameed A.K., Shalal A.I., Kareem S.S.A., Majeed M.A., Humidy S.T. (2021). Internet Addiction and Its Relationship to Gender, Depression and Anxiety Among Medical Students in Anbar Governorate-West of Iraq. Int. Q. Community Health Educ..

[44] Mazidi A.K., Rahimnia F., Mortazavi S., Lagzian M. (2020). Cyberloafing in public sector of developing countries: Job embeddedness as a context. Pers. Rev..

[45] Allan B.A., Dexter C., Kinsey R., Parker S. (2018). Meaningful work and mental health: Job satisfaction as a moderator. J. Ment. Health.

[46] Young K.S., De Abreu C.N. (2011). Internet Addiction. A Handbook and Guide to Evaluation.

[47] Satuf C., Monteiro S., Pereira H., Esgalhado G., Afonso R.M., Loureiro M.J.D.S. (2018). The protective effect of job satisfaction in health, happiness, well-being and self-esteem. Int. J. Occup. Saf. Ergon..

